# Self-reported dietary supplement use in deployed United States service members pre-deployment *vs*. during deployment, Afghanistan, 2013–2014

**DOI:** 10.1186/s40779-017-0141-6

**Published:** 2017-10-26

**Authors:** Shawn M. Varney, Patrick C. Ng, Crystal A. Perez, Allyson A. Araña, Edwin R. Austin, Rosemarie G. Ramos, Vikhyat S. Bebarta

**Affiliations:** 1 University of Texas Health Science Center San Antonio, 7709 Floyd Curl Dr, San Antonio, TX 78229 USA; 2 San Antonio Military Medical Center, 3551 Roger Brooke Drive, San Antonio, TX 78234 USA; 3 Rocky Mountain Poison and Drug Center, Denver Health and Hospital Authority, Denver, CO USA; 4 U.S. Army Institute of Surgical Research, USAF En Route Care Research Center, 3698 Chambers Pass STE B, JBSA Ft Sam Houston, San Antonio, TX 78234 USA; 5 Department of Emergency Medicine, University of Colorado School of Medicine, 13001 E 17th Place, Aurora, CO 80045 USA

**Keywords:** Military, Dietary supplement, Workout, Exercise, Adverse events

## Abstract

**Background:**

Dietary supplement use (protein/amino acids, weight-loss supplements, performance enhancers) is common among U.S. military members. Reported dietary supplement use in deployed troops is limited and is of concern in settings where troops are exposed to high ambient temperatures, increased physical demands, and dehydration. Our objective was to describe dietary supplement use and adverse events (AEs) among deployed U.S. service members compared with their pre-deployment use.

**Methods:**

We conducted an institutional review board (IRB) approved, descriptive study in Afghanistan using a written questionnaire and collected demographic information, dietary supplement use before and during deployment, AEs associated with supplement use, and physical workout routines. Participants were U.S. military personnel of all branches of service deployed to Afghanistan. They were recruited in high-traffic areas in the combat theater. We analyzed the data with descriptive statistics. Paired *t*-test/Wilcoxon signed-rank test was conducted to examine the before/during deployment changes for continuous data, and McNemar’s chi-square test was conducted for categorical data. We constructed separate logistic regression models to determine the best predictors of increases or decreases in dietary supplement use, with demographic information, reasons for using supplements, and education requested/received as covariates in each model. All statistical tests were two-sided at a significance level of 5% (*P* < 0.05).

**Results:**

Data were collected on 1685 participants. Ninety-seven of the participants were in the Army or Air Force.

The participants were more likely to work out daily or more than once a day during deployment. Thirty-five percent of the participants reported no supplement use before or during deployment. The remaining 65% of participants reported increased use and increased frequency of use of supplements (e.g., daily) during deployment compared with pre-deployment. Additionally, more people followed label instructions strictly during deployment *vs*. pre-deployment.

Overall, the frequency of self-reported AEs among supplement users remained consistent before and during deployment. The only significant difference noted was in problems falling or staying asleep, which increased during deployment. In the adjusted logistic regression models, the level of formal education, military branch, occupational specialty, education about dietary supplements, and certain reasons for using supplements (to boost energy, lose weight, gain muscle strength and mass, and as a meal replacement) were significant predictors of changes in supplement use.

**Conclusion:**

Deployed U.S. service members were more likely to use dietary supplements, use more than one supplement and use supplements more frequently during deployment than pre-deployment. No serious AEs were reported, but problems falling or staying asleep increased during deployment.

**Electronic supplementary material:**

The online version of this article (10.1186/s40779-017-0141-6) contains supplementary material, which is available to authorized users.

## Background

Dietary supplement use (protein/amino acids, weight-loss agents, performance enhancers) is common in the United States (U.S.) general population and among U.S. military service members; deployment of service members may be related to dietary supplement use habits [1, 2]. Dietary supplements compose a 32 billion dollar industry, with over 50% of the American public regularly using dietary supplements [3, 4]. Similarly, 53% of U.S. Army soldiers use supplements at least weekly, and other studies using survey data have determined that the overall prevalence of dietary supplements in the military population continues to rise [5–7]. The use of dietary supplements by other country’s armed forces increases during deployment, and the consumption of various nutritional supplements may have AEs such as psychosis [8, 9].

*Dietary supplement* is a broad term that comprises many substances. Congress defined *dietary supplement* in the Dietary Supplement Health and Education Act (DSHEA) of 1994 as a product taken by mouth that contains a “dietary ingredient” intended to supplement the diet [10]. *Dietary ingredients* include vitamins, minerals, herbs, botanicals, amino acids, and substances such as enzymes, organ tissues, glandulars, and metabolites. In addition, dietary supplements can be extracts or concentrates.

We defined dietary supplements as any vitamin, mineral (e.g., multivitamins, magnesium, and calcium), herb (e.g., herbal/homeopathic supplements such as ginkgo and fish oil.), protein (e.g., protein/amino acids such as whey isolate and MuscleMilk®), energy drinks, weight-loss product (e.g., Hydroxycut®, Sensa®, green tea extract, and added fiber), or energy supplements (e.g., N.O.-XPLODE®, C4 by Cellucore®, and Jack3d). We did not include coffee, caffeinated soft drinks, or rehydration sports drinks (e.g., Gatorade).

Prior studies suggest that deployed U.S. service members increase their use of dietary supplements compared to their use in garrison [2, 11]. Dietary supplements have been associated with life-threatening AEs including but not limited to hepatic injury, stroke, and death [1, 11–16]. The increased use of dietary supplements may have negative health effects on our deployed service members. This may have direct implications on the duties of our service members and their ability to perform tasks that affect the mission. The prevalence and patterns of dietary supplement use in garrison and in the deployed setting have not been well characterized. There are limitations inherent in collecting such data.

Our primary objective was to compare U.S. service member dietary supplement use prevalence and patterns pre-deployment vs. during deployment. The secondary objective was to report on any AEs that may be related to dietary supplement use.

## Methods

We conducted a prospective, observational study comparing dietary supplement use and associated AEs pre-deployment and during deployment. The study occurred at Bagram Air Base, Afghanistan from November 2013 to March 2014 and was approved by the U.S. Medical Research and Materiel Command Institutional Review Board.

Study participants were active duty U.S. military personnel from the Air Force, Army, Navy, and Marine Corps who were deployed to Afghanistan at the time. We collected data using an anonymous, 28-item, written questionnaire with fixed responses. Participation was voluntary, and no protected health information was collected. Questions were piloted on a panel of emergency physicians, nurses, research nurses, research personnel, administrative workers, and enlisted troops to review the questionnaire for readability, comprehension, and clarity. Their input led to changes in format, question content, and possible answers as found in the final questionnaire version (Additional file 1: Appendix 1).

Data collected included demographics, medical history, reasons for dietary supplement use (energy boost, muscle building, weight gain/loss, health promotion, performance enhancement, and fat burning), and source of education on supplements. We grouped respondents into three military occupational specialty (MOS) categories: Combat Arms (Artillery, Combat Aviation, Infantry, Special Forces, etc.); Combat Support (Engineers, Military Intelligence, Military Police, Support Aviation, etc.); and Combat Service Support (Chaplain, Finance, Medical, Transportation, etc.). We did not inquire about specific tasks or daily schedules. We also obtained information on routines before and during deployment for workout regimens, dietary supplement use, and AEs associated with supplement use.

Trained research assistants were members of the Joint Combat Casualty Research Team, a team of active duty registered nurses led by a physician assigned to execute research projects in the combat theater. The study authors trained the Research Team in a 90-min orientation session prior to deployment and maintained predetermined, regular contact throughout the study period. The research assistants were given a script for approaching and inviting participants to respond to the survey questions. Questionnaires contained a cover sheet explaining the purpose and the voluntary and anonymous nature of the study.

A convenience sample was recruited in high-traffic areas — namely, the fitness centers, gyms, dining halls, waiting room/noncritical care areas of the emergency department, and the airport passenger terminal for service members returning home. Research assistants distributed and collected the surveys from participants at all hours in the emergency department and from a convenience sample (during the hours of 0700 – 2200) at other locations from November 2013 through March 2014. In this single encounter, the participants reported on their dietary supplement use both pre and during deployment with the use of the survey.Some surveys were incomplete. For these incomplete surveys, we included the questions that were answered in our analysis. We did not compensate participants for participation in the study.

In this study, we defined a dietary supplement user as anyone who ingests substances described in the DSHEA with the intent to complement the diet. We defined an AE as any self-reported, unwanted effect associated with dietary supplement use, especially if it resulted in seeking medical care. *Deployment* was defined as any current or past event or activity that related to duty in the armed forces in an operational area different from the military member’s normal duty assignment [17].

Demographic and survey questions were reported as the means (±SD) for normal continuous variables, medians for non-parametric continuous data, and frequencies and percentages for categorical data, where SD is standard deviation. Paired *t*-test/Wilcoxon sign-rank test was conducted to examine the before/after deployment changes for continuous data, and McNemar’s chi-square test was conducted for categorical data. We also grouped participants based on their change in supplement use from before to during deployment. Those who responded with a higher frequency of use during deployment compared with that before deployment were placed in the increased use category, whereas those who reported lower frequency of use during deployment were placed in the decreased use group. Participants who reported the same frequency of use before and during deployment were categorized in the no change group. We constructed separate logistic regression models to determine the best predictors of increases or decreases in dietary supplement use, with sex, age, race, education, military branch, rank, deployment length, MOS, reasons for using supplements, education requested/received, and education quality as covariates in each model. All statistical tests were two-sided at a significance level of 5% (*P*-value, *P* < 0.05). We used JMP version 13 (Cary, NC, USA) for all statistical analyses.

## Results

Of 1700 individuals recruited, 15 were excluded due to ineligibility (i.e., were not active duty military personnel deployed to Afghanistan). Of the 1685 eligible participants, 79% were male, and approximately 74% were between the ages of 17 and 34 years. Most respondents (97%) were Army and Air Force and were junior and senior enlisted personnel. The majority of participants had been deployed for 6 months or fewer. Regarding military occupational specialty, most participants served as combat support or combat service support (Table 1). Among those with medical problems, the most commonly reported were headaches (34%), hypertension (33%), anxiety (26%), and depression (20%).

**Table 1:** Participant characteristics

Characteristic	Count (%)
Sex	
Male	1324 (79%)
Female	340 (20%)
No answer	21 (1%)
Age (years)	
17-24	469 (28%)
25-34	773 (46%)
35-44	306 (18%)
45-54	103 (6%)
> 54	15 (1%)
No answer	19 (1%)
Race	
Caucasian	922 (55%)
African American	308 (18%)
Hispanic	254 (15%)
Asian	84 (5%)
Other	96 (6%)
No answer	21 (1%)
Education	
High school diploma	243 (14%)
Some college	805 (48%)
Bachelor’s Degree	434 (26%)
Master’s/Ph.D.	183 (11%)
No answer	20 (1%)
Branch of service	
Army	1063 (63%)
Air Force	575 (34%)
Navy	23 (1%)
Marines	5 (<1%)
Other	2 (<1%)
No answer	17 (1%)
Rank	
Officer	317 (19%)
Senior Enlisted	700 (42%)
Junior Enlisted	658 (39%)
No answer	10 (<1%)
Deployment length (months)	
< 1	202 (12%)
1-3	503 (30%)
4-6	501 (30%)
7-9	383 (23%)
> 9	90 (5%)
No answer	6 (<1%)
Military occupational specialty	
Combat arms	250 (15%)
Combat support	661 (39%)
Combat service support	741 (44%)
No answer	33 (2%)

Percentages given are out of all study participants (*n* = 1685)

Compared with pre-deployment routines, participants were more likely to work out daily or more than once a day during deployment. Additionally, the percentage of those who worked out less than twice a week or 2 to 6 times a week decreased during deployment. The number of respondents who exercised for 61-120 min or more than 120 min per session increased during deployment, whereas the number who exercised for 30-60 min or less than 30 min decreased. Nearly twice as many participants lifted weights during deployment compared with before deployment (Table 2).

**Table 2:** Comparison of exercise frequency, duration, and type before and during deployment

Exercise descriptions	Before deployment	During deployment	*P*
Exercise frequency			
Could not work out	27 (2%)	24 (1%)	0.6617
Less than 2 times a week	218 (13%)	139 (8%)	< 0.0001
2-6 times a week	996 (60%)	859 (53%)	< 0.0001
Daily	358 (21%)	503 (31%)	< 0.0001
More than once a day	67 (4%)	109 (7%)	< 0.0001
Exercise duration			
Less than 30 min	133 (8%)	85 (5%)	< 0.0001
30-60 min	999 (61%)	833 (52%)	< 0.0001
61-120 min	456 (28%)	615 (39%)	< 0.0001
More than 120 min	39 (3%)	69 (4%)	< 0.0001
Exercise type			
Weightlifting	669 (40%)	1234 (73%)	< 0.0001
Cardio	1353 (80%)	1300 (77%)	0.0009
Circuit training	402 (24%)	426 (25%)	0.1456
Walking	161 (10%)	185 (11%)	0.0153
Calisthenics	132 (8%)	120 (7%)	0.2008
Other	66 (4%)	42 (2%)	0.0013

Percentages given are out of all study participants (*n* = 1685). Participants may report more than one type of exercise. All *P*-values are for McNemar’s chi-square test for paired proportions

Approximately 35% of respondents (*n* = 595) reported no supplement use before and during deployment; the 1090 remaining participants were included in the following analyses. Frequent supplement use (4 to 6 times a week, daily, or more than once a day), as well as the use of multiple supplements at once, increased during deployment. The use of protein/amino acid supplements and performance enhancers also increased during deployment. Fewer respondents reported following label use strictly (i.e., every time) pre-deployment vs. during deployment, and fewer participants requested and received education about dietary supplements during deployment (Table 3). The most common sources of education were the Internet (24%), health care providers (i.e., medically qualified personnel) (17%), magazines (17%), and friends (16%).

**Table 3:** Comparison of supplement use and adverse events before and during deployment

Supplement characteristics	Before deployment	During deployment	*P*
Supplement use frequency			
Few times a month	97 (9%)	65 (6%)	0.0015
2-3 times a week	195 (18%)	132 (12%)	<0.0001
4-6 times a week	198 (18%)	258 (24%)	<0.0001
Daily	324 (30%)	435 (40%)	<0.0001
More than once a day	58 (5%)	99 (9%)	<0.0001
Any use (any of the above)	895 (82%)	1004 (92%)	<0.0001
No use	201 (18%)	88 (8%)	<0.0001
No answer	17 (2%)	13 (1%)	–
Supplement type			
Vitamins/minerals	628 (58%)	641 (59%)	0.3840
Herbal/homeopathic	243 (22%)	243 (22%)	0.9999
Protein/amino acids	560 (51%)	711 (65%)	<0.0001
Energy drinks	264 (24%)	273 (25%)	0.3608
Weight loss	169 (16%)	184 (17%)	0.1394
Performance enhancers	331 (30%)	416 (38%)	<0.0001
Other	14 (1%)	19 (2%)	0.1317
Use multiple supplements	599 (55%)	684 (63%)	<0.0001
Always followed label instructions	447 (41%)	515 (47%)	<0.0001
Requested education^a^	770 (71%)	277 (25%)	0.0027
Received education^a^	585 (54%)	460 (42%)	<0.0001
Reasons for using supplements			
Boost energy	383 (35%)	460 (42%)	<0.0001
Enhance performance	346 (32%)	427 (39%)	<0.0001
Improve health	493 (45%)	519 (48%)	0.0474
Improve mental health	110 (10%)	123 (11%)	0.0526
Decrease fat	179 (16%)	227 (21%)	<0.0001
Lose weight	174 (16%)	227 (21%)	<0.0001
Gain muscle strength	444 (41%)	588 (54%)	<0.0001
Gain muscle mass	310 (28%)	452 (41%)	<0.0001
Meal replacement	101 (9%)	140 (13%)	<0.0001
Other	20 (2%)	21 (2%)	0.7630
Adverse events			
Headache	33 (3%)	39 (4%)	0.2888
Dizziness, light-headedness	31 (3%)	38 (3%)	0.2230
Tachycardia	92 (8%)	94 (9%)	0.8137
Nervousness	21 (2%)	22 (2%)	0.8185
General weakness	5 (<1%)	6 (<1%)	0.7389
Abdominal pain	10 (1%)	11 (1%)	0.7815
Nausea or vomiting	12 (1%)	19 (2%)	0.1083
Chest pain	5 (<1%)	7 (<1%)	0.7055
Confusion	3 (<1%)	4 (<1%)	0.3173
Problems falling or staying asleep	61 (6%)	79 (7%)	0.0181
Skin changes	8 (<1%)	10 (1%)	0.4142
Other	12 (1%)	17 (2%)	0.1655

Participants who reported no supplement use before AND during deployment (*n* = 595, 35% of total sample) are excluded from the above analyses. Percentages given are out of *n* = 1090. Participants may report more than one supplement type, reason for use, and adverse event. All p-values are for McNemar’s chi-square test for paired proportions

^a^Education or information on supplement use was in the form of the Internet, health care providers, magazines and peers

Participants’ reasons for using dietary supplements also changed from before deployment to during deployment. More respondents cited a desire to boost energy, enhance performance, improve health, decrease fat, lose weight, gain strength, gain muscle mass, and used supplements as meal replacements during deployment. More people followed label instructions strictly during deployment vs. pre-deployment. Overall, the frequency of self-reported AEs among dietary supplement users remained consistent before and during deployment. The only significant difference noted was in problems falling or staying asleep, which increased during deployment (Table 3). Finally, there was no difference in the number of AEs between those who complied strictly with manufacturers’ instructions vs. those who did not.

Over half of the participants who reported supplement use (*n* = 561) reported no change in supplement use, with 266 of those participants maintaining daily use throughout the study. Approximately 12% (*n* = 126) decreased use; of these participants, 84 discontinued supplement use during deployment. Approximately 35% (*n* = 381) increased their use of dietary supplements, with 165 increasing their use to once a day during deployment.

In the adjusted logistic regression model, the odds of decreasing supplement use were higher for participants with advanced degrees (Master’s/PhD) than those for participants with only high school diplomas. Respondents who requested or received education about dietary supplements before deployment were 3 times more likely to report a decrease in supplement use compared to those who did not request or receive education before deployment. Additionally, the odds of decreasing supplement use were lower for participants who cited a desire to boost energy, improve health, lose weight, and gain muscle strength and mass as reasons for using supplements during their deployment. The odds of increasing supplement use were higher for Air Force than for Army. Participants who requested and received education during deployment were twice as likely to report an increase in supplement use than did those who did not. The odds of increasing supplement use were also twice as high for respondents who used supplements to gain muscle strength and as meal replacements. Sex, age, race, rank, military occupational specialty, and deployment length were not significant predictors of reported decreases or increases in dietary supplement use in adjusted logistic regression models (Table 4).

**Table 4:** Adjusted logistic regression models predicting changes in supplement use [OR (95% CI)]

Predictor variables	Decrease use	Increase use
Sex (vs. female)		
Male	0.99 (0.46-2.14)	1.07 (0.63-1.83)
Age (vs. 34 or older)		
17-34	1.36 (0.62-3.00)	1.41 (0.84-2.40)
Race (vs. Caucasian)		
African American	1.19 (0.50-2.85)	1.10 (0.63-1.91)
Hispanic	1.77 (0.76-4.13)	1.01 (0.59-1.74)
Asian/other	1.02 (0.37-2.87)	0.68 (0.36-1.28)
Education (vs. high school diploma)		
Some college	0.93 (0.29-2.96)	0.86 (0.47-1.58)
Bachelor’s degree	1.45 (0.40-5.17)	0.80 (0.39-1.63)
Master’s/PhD	3.32 (1.17-9.40)	0.72 (0.26-1.99)
Branch (vs. Army)		
Air Force	0.80 (0.39-1.63)	1.75 (1.12-2.75)
Navy, Marines, or other	5.16 (0.53-49.98)	0.59 (0.09-3.96)
Rank (vs. officer)		
Junior Enlisted	2.01 (0.68-5.93)	0.66 (0.33-1.32)
Senior Enlisted	1.82 (0.69-4.83)	0.62 (0.32-1.20)
Deployment length (vs. 0-3 months)		
4-6 months	0.58 (0.26-1.29)	0.99 (0.63-1.56)
> 6 months	0.77 (0.32-1.87)	0.86 (0.51-1.44)
Military Occupational Specialty (vs. combat arms)		
Combat support	0.66 (0.21-2.07)	1.59 (0.87-2.91)
Combat service support	0.42 (0.13-1.30)	1.03 (0.56-1.90)
Requested education		
Before deployment	3.47 (1.66-7.26)	0.40 (0.23-0.68)
During deployment	0.40 (0.15-1.07)	2.43 (1.42-4.16)
Received education		
Before deployment	3.28 (1.09-9.90)	0.16 (0.09-0.26)
During deployment	0.58 (0.27-1.23)	1.95 (1.24-3.06)
Sufficient education received	0.58 (0.18-1.83)	0.78 (0.39-1.55)
Reasons for using supplements (during deployment)		
Boost energy	0.31 (0.13-0.69)	0.96 (0.64-1.46)
Enhance performance	0.46 (0.19-1.16)	1.24 (0.79-1.93)
Improve health	0.11 (0.05-0.24)	0.73 (0.48-1.11)
Improve mental health	3.78 (1.00-14.32)	0.98 (0.54-1.80)
Decrease fat	1.61 (0.57-4.55)	0.91 (0.54-1.52)
Lose weight	0.18 (0.04-0.81)	1.47 (0.85-2.54)
Gain muscle strength	0.36 (0.16-0.82)	1.81 (1.11-2.94)
Gain muscle mass	0.33 (0.12-0.88)	1.34 (0.85-2.12)
Meal replacement	0.28 (0.06-1.35)	1.92 (1.06-3.11)

Odds ratio (OR) with 95% confidence intervals (CI) were used

Participants who reported no supplement use before and during deployment (*n* = 595, 35% of total sample) and those who were missing at least one response on frequency of supplement use (*n* = 22, 1% of total sample) were excluded from the above analyses. Some demographic categories have been combined due to low counts

## Discussion

In our study, we found that a greater number of military personnel during deployment vs. pre-deployment reported the following: 1) using dietary supplements; 2) using dietary supplements more frequently (daily/weekly); 3) using multiple supplements; and, 4) experiencing sleep disturbance.

Strengths of our study include adequate power, multiple comparisons assessing deployment factors on dietary supplement use patterns not previously described, and the fact that the study was performed in theater to limit recall bias.

In a 2015 article, Paisley reported on supplement use in military paratroopers and concluded that supplement use occurred at a higher rate among paratroopers who were deployed than among those in garrison [6]. Unlike our study, Paisley did not include data collected on a general active duty population in which we found a general increase in supplement use among the three MOSs [6].

Austin et al. [11] conducted a study that described the dietary supplement use trends in the U.S. Army vs. the civilian population. In this article, Austin et al. reported that supplement use by soldiers increased greater than 5% compared with the use in the civilian population between 2006 and 2007 and 2010-2011 [7]. In another 2016 article, Austin et al. reported on a study using a survey to characterize dietary supplement use by soldiers in garrison and in a combat zone. Austin et al. found that deployed soldiers were more likely to use protein, amino acids and combination products than were garrison soldiers. Most of their data came from surveys collected from garrison soldiers (garrison *n* = 1001, deployed *n* = 221) [11]. This differs from our study in which all of the data were collected from deployed troops. There may be differences in troops who deploy and those who do not, and this may have implications on the data reported. A more effective study would require a daily log of supplement use before and during deployment. Short of this, future studies could survey troops who are going to deploy, and then use the same survey on those troops at the end of their deployment.

In a recent report by the Military Health System Communications Office, military health experts warned about the dangers of supplement use, particularly with a practice known as “stacking,” where people use multiple supplements simultaneously [12]. In our data we report on the increased use of multiple supplements while deployed. This may indicate that military members are more likely to practice “stacking” while deployed. Little is known about health outcomes after combining multiple supplements, and further studies are needed to understand the implications of this practice.

Previous studies report on AEs associated with dietary supplements. In 2015, Geller et al. estimated that 23,005 emergency department visits per year were attributed to AEs related to dietary supplements [13]. As in our study, Geller defined AEs events to include palpitations, chest pain, and tachycardia, but Geller did not characterize these AEs specifically in the military population. In contrast, we found surprisingly few serious AEs. Plausible explanations include that some military members may not have reported AEs for fear of negative repercussions from leadership, even though the questionnaires were anonymous. Furthermore, people who had serious AEs may have been previously evacuated from the theater and may not have been available to participate in this survey.

Another possibility is compliance with labels. In our study, there were dietary supplement users who reported strictly complying with the label instructions regarding number and size of servings per workout or per day. This has not been described in previous studies and is an important finding that may explain the low AE rate in our population. In addition, it is consistent with, and may have resulted from, the increased education service members reported receiving pre-deployment. Participants may have anticipated an increase in use of dietary supplements while deployed and may have taken initiative to learn more about them. Participants reported seeking dietary supplement education from a variety of sources including but not limited to the Internet, health care providers, and magazines. The accuracy of information from these sources is not known. It is also unknown if participants used Operation Supplement Safety as a reference, which serves as a resource for military members on the safety of supplement use^17^. As dietary supplement use by military members continues to increase in general, and in deployed service members in particular, educational materials regarding responsible use of dietary supplements should continue to be offered as part of pre-deployment training.

Unlike some studies, severe AEs such as liver injury, intracranial bleeding and severe chest pain were not reported in our study [9, 13–16]. While the exclusion of any potentially unstable hospital patients from study participation may have resulted in some missed cases, our findings offer some alternative explanations for this unchanged AE rate. Service members were more likely to exercise daily and for longer sessions during deployment. The majority of these increases came from weight lifting rather than cardiovascular training as has been previously documented [18]. Previous studies have shown that body composition and physical fitness performance changes occur in service members who have been deployed to Iraq and Afghanistan [18, 19]. Such changes include improved performance in strength exercises and a decline in aerobic performance; however, the overall impact of these findings on the mission and productivity in the deployed setting remains uncertain [18–20]. Correspondingly, the supplements responsible for the significant increase in our primary outcome were categorized as either protein/amino acids or performance enhancers. Protein/amino acid supplements have generally been found to be safe, unlike weight-loss supplements that have been associated with a multitude of reported AEs even after the banning of ephedrine alkaloids in such stimulants [13]. It is possible that any greater risk to the study population from increased performance enhancer usage may have been offset by no increase in the use of weight-loss supplements. In addition, the setting in which the study occurred should be considered. High ambient temperature and increased activity that may be associated with training have been reported in the deployed setting and can have physiological implications. In future studies, this issue should be addressed further.

Our findings, along with data from other studies mentioned, can help further understand our service members’ supplement practices while deployed and complement discussions that warn about the potential dangers of supplement use [1, 8, 9, 12–16]. In addition, the environment of the deployed setting may contribute to sleep disturbances as well. Future studies are needed to further understand supplement use practices in the deployed setting and to determine the true prevalence of reported AEs. This information can help guide policy and educational guidelines on which supplements may or may not be harmful to our service members.

Sleeping problems (i.e., falling or staying asleep) were reported as a significant AE during deployment. We did not ask for further details and do not know if this was in a particular sex, age, race, rank, or MOS classification. In addition, it is unknown which supplements the individuals who reported sleep problems may have taken, if any. Some may argue that an increased use of energy drinks, including greater caffeine intake, would disrupt sleep, while others may suggest that the greater caffeine intake helps maintain alertness during the high operational tempo that restricts sleep. In our study we did not ask specifically about coffee or caffeinated soft drinks. Furthermore, self-reported energy drink consumption did not increase during deployment. It is likely that other factors such as ambient noise, environmental conditions, lodging arrangements and facilities, demanding physical duties, and stressful conditions, contributed to sleeping problems. Although the impact on sleep from dietary supplement intake is unknown, it is clear that increased dietary supplement use was more common among those with sleeping problems.

Although previous surveys of dietary supplement use in deployed environments have considered baseline education levels of the participants, ours is the first to assess the impact of dietary supplement-specific education on prevalence of use. Respondents who reported receiving education about dietary supplements before deployment were more likely to decrease supplement use, whereas those receiving dietary supplement education while deployed reported increasing use. Our results cannot easily explain this divergence, but its existence may be important in future dietary supplement education. Several possibilities exist. It is possible that pre-deployment education came from more reputable sources, such as healthcare professionals or regulated Internet sites such as Operation Supplement Safety (OPSS), whereas during deployment, education may have come more from friends or unregulated online sources such as message boards. Additionally, while we asked respondents about reasons/goals for supplement use, we did not ask specifically if they sought dietary supplement education before deployment for these same reasons, or if their goals changed due to the education they had received. For instance, perhaps our population did not increase weight-loss supplement use in particular while deployed due to education they had received pre-deployment. In addition, perhaps respondents who received education before deployment were motivated by greater safety, while those who received education during deployment sought greater effectiveness in meeting predetermined goals.

Our study is also the first to consider an advanced degree (Master’s/PhD) as a separate category. Compared with the advanced degree group, all other education groups were less likely to decrease their use of dietary supplements during deployment. Previous studies have found increased dietary supplement use among military members with greater formal education both in garrison [21] and in the deployed setting [11]. However, in those studies, military members with advanced degrees were grouped alongside members holding only bachelor’s degrees. Future studies could evaluate whether military members with education beyond a bachelor's degree decrease their dietary supplement use in general compared with others in garrison or deployed, or if the high number of medical personnel at Bagram Air Field had an effect on our results.

### Limitations

Our study has several limitations. First, the major objective of comparing supplement use pre- and during deployment relies upon a combination of point prevalence and recall data. However, the reality is that real-time pre- and during-deployment data would be almost impossible to collect in sufficient numbers in an operational military population. Second, we collected data using a self-reported survey for dietary supplement use patterns, and we could not verify the reports of disease or illness in the medical record. Third, there may have been recall bias for pre-deployment use patterns. Additionally, some surveys had incomplete data, and the number of non-responders varied between the pre- and during-deployment groups. Next, although the questionnaires were anonymous, some participants may not have reported AEs for fear of adverse actions from leadership. Regardless, the IRB would not permit the study to occur without anonymity. Furthermore, people suffering serious AEs may have been evacuated from the theater, thus eliminating them from study participation. Additionally, we did not ask about steroid intake or illicit stimulants, nor did we specify which dietary supplement(s) individuals were taking. It would be interesting to identify any particular supplements that are more prevalent in this study population and/or to isolate if certain supplements are associated with any bad outcomes compared with others. Next, we studied dietary supplements that are available over the counter. It may be beneficial to include prescription supplements in future studies to get a more comprehensive understanding of dietary supplement use [22, 23]. Recent manuscripts have discussed biomarkers as an approach to monitor any AEs of dietary supplements [22]. Measuring these markers, if validated, may demonstrate more utility in identifying if supplements have AEs on our service members. Lastly, there are potential confounders inherent to the deployed setting that may have caused sleep disturbances in the deployed setting for which we did not account.

## Conclusions

In a questionnaire study, deployed U.S. service members anonymously reported using dietary supplements more commonly, more frequently, and using multiple supplements at once while deployed compared with pre-deployment. Of the AEs studied, sleep disturbance increased during deployment.

## Additional file

Additional file 1: Appendix 1. Dietary supplement use in Among Deployed United States Service Members Questionnaire. (DOCX 38 kb)

## Abbreviations

AE: Adverse event
ED: Emergency department
IRB: Institutional review board
MOS: Military Occupational Specialty
U.S.: United States
