# Supplementary material for: Self-reported dietary supplement use in deployed United States service members pre-deployment vs. during deployment, Afghanistan, 2013–2014
Source: Mil Med Res. 2017 Oct 26;4:34. doi: 10.1186/s40779-017-0141-6 (PMC5657096; doi:10.1186/s40779-017-0141-6)
Supplement: Additional file 1: — Appendix 1. Dietary supplement use in Among Deployed United States Service Members Questionnaire. (DOCX 38 kb) [file 40779_2017_141_MOESM1_ESM.docx]

**Dietary supplement use in Among Deployed United States Service Members Questionnaire**

**Please do NOT put your name or Social Security# on this sheet**

Congress defined the term **"dietary supplement"** in the Dietary Supplement Health and Education Act (DSHEA) of 1994 as “a product taken by mouth that contains a ‘dietary ingredient’ intended to supplement the diet.” This is a broad definition that includes many products, even natural substances. (See table.) Dietary supplements are found in many forms such as tablets, capsules, softgels, gelcaps, liquids, powders, or bars. (For the purpose of this study we do not consider Gatorade or any other power drink to be a dietary supplement.)

Common dietary ingredients that supplement the diet.

| Vitamins | Amino acids | Metabolites |
| --- | --- | --- |
| Minerals | Enzymes | Extracts |
| Herbs | Organ tissues | Concentrates |
| Botanicals (“natural products”) | Glandulars (from animal glands; i.e., adrenals, etc.) | Caffeine (other than coffee, tea) |

**DEMOGRAPHICS:**

1. What is your age group?
   1. 17-24 years
   2. 25-34 years
   3. 35-44 years
   4. 45-54 years
   5. Over 55 years
2. What is the highest level of education you have earned?
   1. High school diploma or equivalent
   2. Some college
   3. College graduate/Bachelor’s degree
   4. Master’s degree / Doctorate degree
3. Gender
   1. Male
   2. Female
4. What is your race/ethnicity?
   1. White/non-Hispanic
   2. Black/non-Hispanic
   3. Hispanic
   4. Asian/Pacific Islander
   5. American Indian
   6. Other ____________________________________________________________
5. Service Branch
   1. Army
   2. Air Force
   3. Navy
   4. Marine
   5. Other ______________________________________________________________________
6. DURING your deployment what is your military status?
   1. Active duty
   2. Activated reservist
   3. Activated guardsman
7. Officer or enlisted?
   1. Enlisted (E1-E4)
   2. Enlisted (E5-E9)
   3. Warrant officer
   4. Commissioned officer
8. What is your occupation while deployed (MOS, Rating, AFSC)?
   1. Combat Arms (Infantry, Artillery, Combat Aviation, Special Forces, etc.)
   2. Combat Support (Engineers, Military Intelligence, Military Police, Support Aviation, etc.)
   3. Combat Service Support (Medical, Transportation, Finance, Chaplain, etc.)
9. How long have you currently been deployed?
10. Less than 1 month
11. 1-3 months
12. 4-6 months
13. 7-9 months
14. > 9 months
15. Do you use tobacco products?
    1. Yes currently
    2. Former use
    3. Never

11a. Have you ever been diagnosed with any of the medical problems listed below?

1. Yes
2. No

11b. If you answered yes, circle all that apply.

1. Anxiety
2. Depression
3. Diabetes
4. Headaches
5. High blood pressure
6. PTSD (post traumatic stress disorder)
7. TBI (traumatic brain injury)
8. Thyroid problems
9. Other _______________________________________________________________

**WORKOUT SCHEDULE:**

12. How often did/ do you work out?

**BEFORE deployment:**

- 1. I could not work out
  2. Less than 2 times per week
  3. 2 -6 times a week
  4. Daily
  5. More than once a day

**DURING deployment:**

- 1. I cannot work out
  2. Less than 2 times per week
  3. 2 – 6 times a week
  4. Daily
  5. More than once a day

13. How long was/is your typical workout?

**BEFORE deployment:**

1. I did not work out
2. < 30 minutes
3. 30-60 minutes
4. 61 – 120 minutes
5. > 120 minutes

**DURING deployment:**

1. I do not work out
2. <30 minutes
3. 30 – 60 minutes
4. 61 – 120 minutes
5. > 120 minutes
6. If you work out, what did/does your workouts consist of?

**BEFORE deployment:**

- 1. Not applicable. I did not work out
  2. Weight lifting (strength training)
  3. Cardio *(running, elliptical, stair climber, etc.)*
  4. Circuit Training *(P90-X, Insanity, CrossFit, etc.)*
  5. Calisthenics *(Zumba, Yoga, Pilates, etc.)*
  6. Walking
  7. Other *(Martial Arts, etc.)*

Please list: ___________________________

**DURING deployment:**

1. Not applicable. I do not work out
2. Weight lifting (strength training)
3. Cardio *(running, elliptical, stair climber, etc.)*
4. Circuit Training *(P90-X, Insanity, CrossFit, etc.)*
5. Calisthenics *(Zumba, Yoga, Pilates, etc.)*
6. Walking
7. Other *(Martial Arts, etc.)*

Please list: ___________________________

**DIETARY SUPPLEMENTS**

**Please answer the following:**

**□** I did not use dietary supplements before deployment.

□ I have **NEVER** used dietary supplements. If you have **NEVER** used dietary supplements, you may **stop** here.

1. If you used/use dietary supplements, which formulations of dietary supplement(s) do you use? (Circle all that apply.)

**BEFORE deployment:**

- 1. I did not use Dietary Supplements
  2. Vitamins/Minerals *(multivitamins, Magnesium, Calcium etc.)*
  3. Herbal/Homeopathic supplements *(Ginkgo, Fish Oil, etc.)*
  4. Protein/Amino Acids *(Whey isolate, MuscleMilk, etc.)*
  5. Caffeine *(Red Bull, Monster, 5-Hour Energy, etc.)*
  6. Weight Loss *(Hydroxycut, Sensa, Green Tea Extract, added Fiber, etc.)*
  7. Performance Enhancers *(Creatine, N.O.-XPLODE, C4, Jack3d, etc.)*
  8. Other _________________________________________________

**DURING deployment:**

1. I do not use Dietary Supplements
2. Vitamins/Minerals *(multivitamins, Magnesium, Calcium etc.)*
3. Herbal/Homeopathic supplements *(Ginkgo, Fish Oil, etc.)*
4. Protein/Amino Acids *(Whey isolate, MuscleMilk, etc.)*
5. Caffeine *(Red Bull, Monster, 5-Hour Energy, etc.)*
6. Weight Loss *(Hydroxycut, Sensa, Green Tea Extract, added Fiber, etc.)*
7. Performance Enhancers *(Creatine, N.O.-XPLODE, C4, Jack3d, etc.)*
8. Other _________________________________________________
9. If you used/use dietary supplements why did you use them? (Please circle all that apply).

**BEFORE delployment:**

1. I did not use dietary supplement(s)
2. To boost my energy
3. To lose weight
4. To decrease fat
5. To gain muscle strength
6. To gain muscle weight
7. To enhance performance
8. To improve my health
9. To Improve my mental health
10. As a meal replacement
11. Other __________________

**DURING deployment:**

1. I do not use dietary supplement(s)
2. To boost my energy
3. To lose weight
4. To decrease fat
5. To gain muscle strength
6. To gain muscle weight
7. To enhance performance
8. To improve my health
9. To Improve my mental health
10. As a meal replacement
11. Other __________________

17. How frequently did/do you use dietary supplements?

**BEFORE deployment**

- 1. I did not use dietary supplements
  2. Few times a month
  3. 2-3 times a week
  4. 4-6 times a week
  5. Daily
  6. More than once a day (including before, DURING, and/or after working out)

**DURING deployment**

1. I do not use dietary supplements
2. Few times a month
3. 2-3 times a week
4. 4-6 times a week
5. Daily
6. More than once a day (including before, during, and/or after working out)

18. How long have you been using supplements?

**BEFORE deployment**

a. I did not use dietary supplements

b. 1-2 month

c. 3-6 months

d. 7-9 months

e. 10-12 months

f. > 12 months

**DURING deployment**

a. I do not use dietary supplements

b. 1-2 month

c. 3-6 months

d. 7-9 months

e. 10-12 months

f. > 12 months

19a. How has your use of dietary supplements changed **DURING** deployment?

1. Not applicable. I do not take dietary supplements
2. No change
3. Decreased
4. Increased
5. Some supplement use increased and some decreased

If Applicable, please specify: ________________________________________________________________________

19b. If you answered that your use of dietary supplements **“increased,”** how much did it increase?

- 1. A little more than usual
  2. Twice as much as usual
  3. 3-4 times as much as usual
  4. 5 or more times as much as usual

20a. Have you ever requested education about dietary supplements?

**BEFORE deployment**

1. I did not take dietary supplements
2. Yes
3. No

**DURING deployment**

a. I do not take dietary supplements

b. Yes

c. No

20b. Have you ever been provided with education about dietary supplements?

**BEFORE deployment**

1. I did not take dietary supplements
2. Yes
3. No

**DURING deployment**

a. I do not take dietary supplements

b. Yes

c. No

20c. If you answered **”YES,”** that you have been provided with education about dietary supplements, who/what was the source of information? Please circle all that apply.

- 1. Healthcare provider
  2. Education class
  3. Mandatory briefing
  4. Discussed with a friend
  5. Magazine article
  6. Internet information
  7. Commercial on TV
  8. Other ______________________________________________________________________

20d. Was the education you received about dietary supplements ____________?

a. Sufficient / adequate

b. Insufficient / inadequate

21. How often do you follow the instructions on the **label exactly** (serving size, frequency, etc.)?

**BEFORE deployment**

1. I did not take dietary supplements.
2. Never
3. Sometimes
4. Usually
5. Almost every time
6. Every time

**DURING deployment**

1. I do not take dietary supplements.
2. Never
3. Sometimes
4. Usually
5. Almost every time
6. Every time
   - 1. If you use the supplement in a different way from the instructions listed on the label, what do you change?

**BEFORE deployment**

a. I did not take dietary supplements

b. Larger serving size

c. Smaller serving size

d. More frequent use

e. Less frequent use

f. Other: _____________________

**DURING deployment**

a. I do not take dietary supplements

b. Larger serving size

c. Smaller serving size

d. More frequent use

e. Less frequent use

f. Other: _____________________

- - 1. Are you getting the results you wanted with dietary supplements use?

**BEFORE deployment**

1. I did not take dietary supplements
2. Yes
3. No

**DURING deployment**

a. I do not take dietary supplements

b. Yes

c. No

24a. Did you have any unanticipated or unwanted results?

**BEFORE deployment**

1. I did not take dietary supplements
2. Yes
3. No

**DURING deployment**

a. I do not take dietary supplements

b. Yes

c. No

24b. If you answered yes, circle all that apply.

**BEFORE deployment**

1. Weight gain (unanticipated or unwanted)
2. Weight loss (unanticipated or unwanted)
3. Other ____________________________

**DURING deployment**

1. Weight gain (unanticipated or unwanted)
2. Weight loss (unanticipated or unwanted)
3. Other ____________________________

25. What **side effects** do you suspect you may have experienced or have attributed to the use of dietary supplement(s?

**BEFORE deployment**

- 1. I did not take dietary supplements
  2. None
  3. Headache
  4. Dizziness, light headed, or faint feeling
  5. Fast heart rate
  6. Nervousness
  7. General weakness
  8. Abdominal pain
  9. Nausea or vomiting
  10. Chest pain
  11. Confusion
  12. Problems sleeping (falling asleep, staying asleep)
  13. Skin changes (color, acne, other)
  14. Other ___________________________

**DURING deployment**

1. I do not take dietary supplements
2. None
3. Headache
4. Dizziness, light headed, or faint feeling
5. Fast heart rate
6. Nervousness
7. General weakness
8. Abdominal pain
9. Nausea or vomiting
10. Chest pain
11. Confusion
12. Problems sleeping (falling asleep, staying asleep)
13. Skin changes (color, acne, other)
14. Other ___________________________

26a. If you had any side effects, did this experience cause you to change your dietary supplements use?

**BEFORE deployment**

1. I did not take dietary supplements
2. I did not have side effects
3. Yes
4. No

**DURING deployment**

1. I do not take dietary supplements
2. I did not have side effects
3. Yes
4. No

26b. If the side effects caused you to change your dietary supplement use, please indicate the change.

**BEFORE deployment**

1. I stopped using supplements permanently
2. I temporarily stopped using supplements

c. I decreased my supplement use

d. I did not change my supplement use routine

**DURING deployment**

a. I stopped using supplements permanently

b. I temporarily stopped using supplements

c. I decreased my supplement use

d. I did not change my supplement use routine

27. If you had **a problem** associated with dietary supplement use, what was the outcome? (Circle all that apply.)

**BEFORE deployment**

1. I did not take dietary supplements
2. Not applicable, I did not have a problem
3. Rested but continued the work out session
4. Stopped the work out session
5. I wanted to get medical attention but waited it out instead
6. I was encouraged by others to get medical care but did not
7. I was encouraged by others to stop taking the supplement but did not
8. I sought medical care and was released
9. I sought medical care and was admitted to the hospital
   1. If you missed work: how many days did you miss work? __________
10. The problem had a negative effect on my work.
11. Other ________________________________________________________________

**DURING deployment**

1. I do not take dietary supplements
2. Not applicable, I did not have a problem
3. Rested but continued the work out session
4. Stopped the work out session
5. I wanted to get medical attention but waited it out instead
6. I was encouraged by others to get medical care but did not
7. I was encouraged by others to stop taking the supplement but did not
8. I sought medical care and was released
9. I sought medical care and was admitted to the hospital
   1. If you missed work: how many days did you miss work? __________
10. The problem had a negative effect on my work.
11. Other ________________________________________________________________

28. How often did/do you use more than one dietary supplement?

**BEFORE deployment**

1. I did not take dietary supplements
2. Never, I take only 1 supplement
3. Occasionally / 1-2 times a MONTH
4. Sometimes / 1-2 times a WEEK
5. Frequently / 3-6 times a week

e. Always / daily

f. More than once a day

**DURING deployment**

1. I do not take dietary supplements
2. Never, I take only 1 supplement
3. Occasionally / 1-2 times a MONTH
4. Sometimes / 1-2 times a WEEK
5. Frequently / 3-6 times a week

e. Always / daily

f. More than once a day

***Thank you for your time and participation!!!***
